# Transcriptome and GWAS Analyses Reveal Candidate Gene for Root Traits of Alfalfa during Germination under Salt Stress

**DOI:** 10.3390/ijms24076271

**Published:** 2023-03-27

**Authors:** Fei He, Tianhui Yang, Fan Zhang, Xueqian Jiang, Xianyang Li, Ruicai Long, Xue Wang, Ting Gao, Chuan Wang, Qingchuan Yang, Lin Chen, Junmei Kang

**Affiliations:** 1 Institute of Animal Science, Chinese Academy of Agricultural Sciences, Beijing 100193, China; 2 Institute of Animal Science, Ningxia Academy of Agricultural and Forestry Sciences, Yinchuan 750002, China

**Keywords:** alfalfa, GWAS, salt stress, root traits, SNP

## Abstract

Alfalfa growth and production in China are negatively impacted by high salt concentrations in soils, especially in regions with limited water supplies. Few reliable genetic markers are currently available for salt tolerance selection. As a result, molecular breeding strategies targeting alfalfa are hindered. Therefore, with the continuous increase in soil salinity in agricultural lands, it is indispensable that a salt-tolerant variety of alfalfa is produced. We collected 220 alfalfa varieties around the world for resequencing and performed genome-wide association studies (GWASs). Alfalfa seeds were germinated in saline water with different concentrations of NaCl, and the phenotypic differences in several key root traits were recorded. In the phenotypic analysis, the breeding status and geographical origin strongly affected the salt tolerance of alfalfa. Forty-nine markers were significantly associated with salt tolerance, and 103 candidate genes were identified based on linkage disequilibrium. A total of 2712 differentially expressed genes were upregulated and 3570 were downregulated based on transcriptomic analyses. Some candidate genes that affected root development in the seed germination stage were identified through the combination of GWASs and transcriptome analyses. These genes could be used for molecular breeding strategies to increase alfalfa’s salt tolerance and for further research on salt tolerance in general.

## 1. Introduction

Salt stress can affect plant productivity, leading to retarded development, with this effect continuing throughout the growth and developmental stages of the plant [1]. One effect of salt stress is reduced water availability and increased osmotic stress imposed on plants, resulting in nutritional disorders and, ultimately, the inhibition of plant growth and photosynthesis [2]. Salt stress in the early stages of plant development can reduce the grain yield and plant protein content, resulting in declines in subsequent plant growth and yields [3,4]. During seed germination, the enzyme activity and water absorption capacity of seeds under salt stress significantly decrease and the germination rate of the seeds rapidly reduces [5]. Therefore, investigating the effect of salt stress in the seed germination stage is important, which is the first step in studying its impact on plant growth and nutrient production [6].

The root system is important in the growth, development, and physiology of crop plants, as well as in their responses to various stresses. The roots absorb water and nutrients from the soil and synthesize plant hormones, making them a necessary synthesis site for plant growth and development [7]. Serving as the interface for plant–soil interactions, the roots also play a key role in responding to environmental changes that can affect important traits, such as salt tolerance [8], drought resistance [9], and resistance to other abiotic stressors [10,11]. Under conditions of salt stress, the first response by plants is elicited by the roots, the functioning of which depends on their characteristics and structure. Reducing the density of lateral roots and the number of axial roots in maize allows for greater axial root elongation and reduces root system competition among plants, thereby improving the water capture and resistance capabilities of maize plants to adverse environments [12]. Therefore, studying the mechanisms employed by plant roots to resist salt stress is essential for expanding our knowledge of plant salt tolerance, which could be used to develop more salt-tolerant crop varieties for improved agricultural sustainability.

Alfalfa (*Medicago sativa* L.) has the characteristics of high yield and high quality; it can improve the soil of saline–alkali land due to its ability to fix atmospheric nitrogen [13]. Alfalfa is easily affected by high-salinity soils, preventing many existing alfalfa varieties from growing on saline lands [14,15]. Therefore, studying the salt tolerance of alfalfa for the development of animal husbandry is important. A surge in the application of genome-wide association studies (GWASs) to a wide variety of plants has occurred in recent years. Many single-nucleotide polymorphisms (SNPs) related to various resistance traits have been identified, including those that confer abiotic stress in maize and soybean plants [16,17,18]. Many SNPs associated with salt and drought tolerance [19,20] and water scarcity [21] have been identified in alfalfa through GWASs. However, the genetics and molecular mechanisms underlying the responses to salt stress in alfalfa remain unclear, thus, greatly limiting the targeted improvement of alfalfa varieties [22,23]. The recent publication of several alfalfa cultivar genomes [13,23,24] may provide a theoretical basis for the identification of salt tolerance genes in alfalfa.

This study involved GWASs based on root traits under normal and salt stress conditions by combining GWASs and RNA-seq analyses to investigate the alleles associated with salt tolerance and candidate genes associated with root system development. The findings of this study might provide valuable molecular markers to improve the molecular breeding strategies for alfalfa.

## 2. Results

### 2.1. Analysis of Phenotypic Variations and Correlations among Salt-Related Root Traits

The following four traits were measured under different salt concentrations: the number of lateral roots (LRs), root length (RL), root volume (RV), and root diameter (RD). The descriptive statistics of the relative values of root traits under a salt treatment were calculated (Table 1). In most traits, the range of phenotypic variation was relatively large among different alfalfa varieties under different salt concentrations, with medians ranging from 0.88 (for RV_200) to 1.12 (for LR_100). RD_200 varied the most, with values ranging from 0.31 to 5.57 and a mean of 1.72. RL_200 varied the least, with values ranging from 0.64 to 1.58 and a mean of 1.06. The coefficient of variation ranged from 0.32 to 0.86.

**Figure 1:**
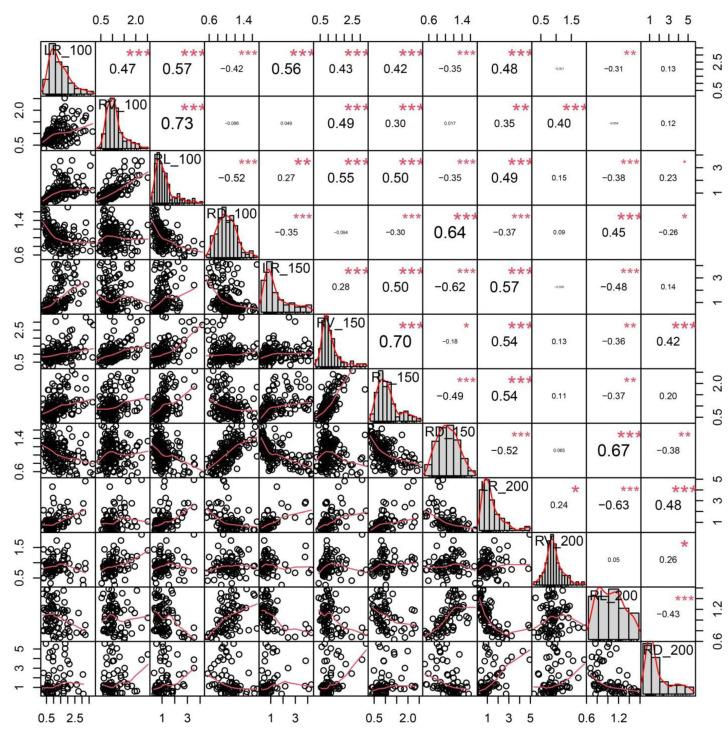
Distributions of and correlations between 12 relative phenotypic traits. The frequency distribution of each trait is shown on a central diagonal in the form of a histogram. Scatter plots between every pair of traits are shown in the areas below the diagonal, and numerical correlation coefficients between every pair of traits are shown in the areas above the diagonal. *, **, and *** indicate significance at *p* < 0.05, *p* < 0.01, and *p* < 0.001, respectively.

In total, four root traits with normal distributions under three salt concentrations were measured (Figure 1). The correlation analysis showed that, for these traits, the largest correlation was between RV_100 and RL_100, reaching 0.73, followed by RV_150 and RL_150, reaching a significance level of 0.70. All other traits displayed significant differences at a salt concentration of 100 mM, excluding RV_100 and RD_100. All four traits showed significant differences at a salt concentration of 150 mM. All other traits exhibited significant differences at a salt concentration of 200 mM, excluding RV_200 and RL_200. The traits LR_200, RL_200, RL_200, and RD_200 exhibited a significant negative correlation. Most of these traits displayed positive correlations under different concentrations of salt treatment.

We divided our population into three categories based on their breeding status. The statistical analysis of these 12 salt-tolerance-related traits showed no significant differences, except for the LR_150 phenotypes of the wild and landrace subgroups, as well as of the wild and cultivar subgroups, for the RV_150 phenotypes of landrace and cultivar subgroups, and for the RV_200 phenotypes of wild and cultivar subgroups. The landrace and cultivars exhibited a greater salt tolerance, whereas the wild varieties had a lower salt tolerance in this population (Figure S1). We also divided our population into four categories based on their geographical origin. In these four subgroups, American and Turkish varieties had a better salt tolerance, followed by European and Chinese varieties (Figure S2).

### 2.2. GWAS and Identification of Candidate Genes for 12 Relevant Phenotypic Traits

**Figure 2:**
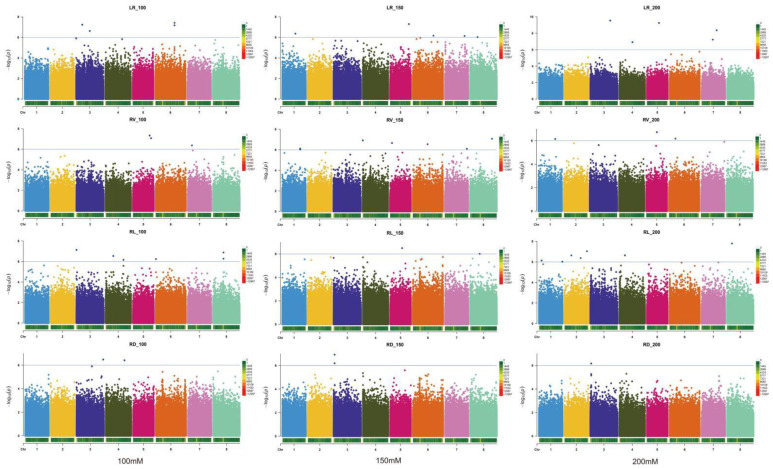
Manhattan plots of marker–trait associations for salt tolerance traits. Significant markers that passed a cutoff log (*p*-value) of 6 are above the dot lines. The GWAS was performed using Bayesian-information and Linkage-disequilibrium Iteratively Nested Keyway C software, and the threshold for significantly associated loci was a logarithm of odds score of ≥6 (blue line). Different colors represent markers on different chromosomes.

A GWAS was conducted on the 12 relative traits (LR_100, RV_100, RL_100, RD_100, LR_150, RV_150, RL_150, RD_150, LR_200, RV_200, RL_200, and RD_200). We identified 49 significant loci (Figure 2 and Table S1). These SNPs were distributed across all chromosomes. They included a maximum of eight SNPs on chromosome three. The fewest number of SNPs, five, was found on chromosomes two and seven. Among the 12 traits, seven significant SNPs associated with RV_150 and nine significant SNPs associated with RL_200 were found. For RV_150, these SNPs were located on six chromosomes, but were not found on chromosomes two and three (Table S1). RL_100 had six significant markers distributed on chromosomes three, four, six, and eight. Only one marker (chr3__3146487) was significantly associated with RD_200.

**Figure 3:**
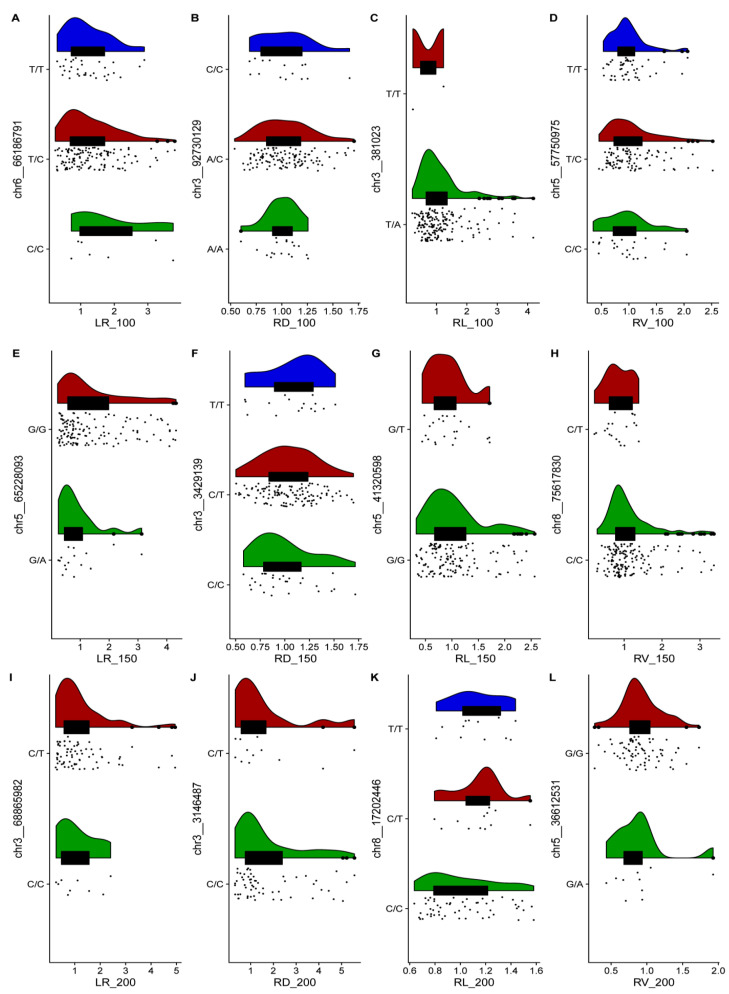
Raincloud plots of the highest distribution of the salt stress of plants with relevant SNP genotypes. The top plots represent the kernel density estimation, the middle plots represent box diagrams, and the bottom plots represent dithering scatter diagrams. Different colors represent different genotypes. (**A**–**D**) represents the genotype of Chr6__66186791, Chr3__92730129, Chr6__381023 and Chr6__57750975 at 100mM salt concentrations. (**E**–**H**) represents the genotype of chr5__65228093, Chr3__3429139, chr5__41320598 and chr8__75617830 at 150mM salt concentrations. (**I**–**L**) represents the genotype of chr3__68865982, chr3__3146487, chr8__17202446 and chr5__36612531 at 200mM salt concentrations.

One SNP with the highest threshold for each trait was selected to construct a raincloud plot so as to observe the effects of different alleles on salt tolerance (Figure 3). No homozygous genotypes (*A*/*A*) existed for SNPs chr3__381023, chr5__65228093, or chr5__36612531. In addition, no homozygous genotypes (*T*/*T*) existed for chr5__41320598, chr8__75617830, chr3__68865982, and chr3__3146487. Therefore, we speculated that the two genotypes might be correlated with root salt tolerance in alfalfa. Next, we identified all genes within the 40 kb range of 49 significant SNP loci based on linkage disequilibrium (LD) and identified 103 candidate genes related to salt tolerance based on the sequence similarity with *A. thaliana* (Table S2).

### 2.3. RNA-Seq Analysis

**Figure 4:**
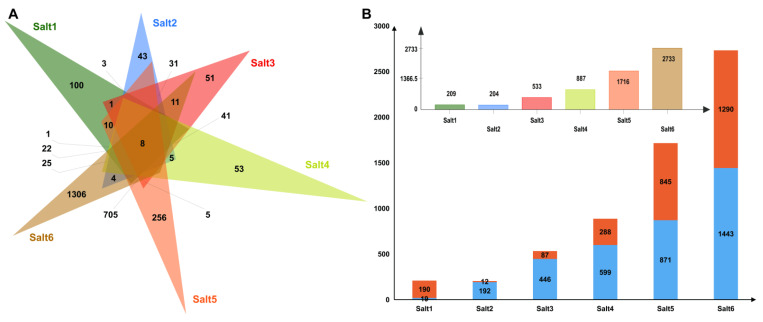
Transcriptomic analysis. (**A**) All DEGs are expressed in different colors in the six different stages of salt stress. (**B**) Upregulated and downregulated DEGs in the six periods. The blue box above represents the downregulated DEGs, and orange represents the upregulated DEGs.

A total of 3479 differentially expressed genes (DEGs) were identified, with 8 genes common in all six stages (Figure 4A). A total of 209, 204, 533, 887, 1716, and 2733 DEGs were found in the six stages (Figure 4B). A total of 190, 12, 87, 288, 845, and 1290 DEGs were upregulated and 19, 192, 446, 599, 871, and 1443 DEGs were downregulated in the Salt1 to Salt6 groups, respectively (Figure 4B and Tables S3–S8). The gene ontology (GO) analysis showed that the largest subcategory was the cellular process in the biological process (Figure S3). For the cellular component, the two largest subcategories were cells and cell parts. The two largest subcategories were catalytic activity and binding in the molecular function category, except for Salt2 for nucleic acid binding transcription factor activity and catalytic activity.

### 2.4. Candidate Gene Analysis Related to Salt Tolerance in Alfalfa

**Figure 5:**
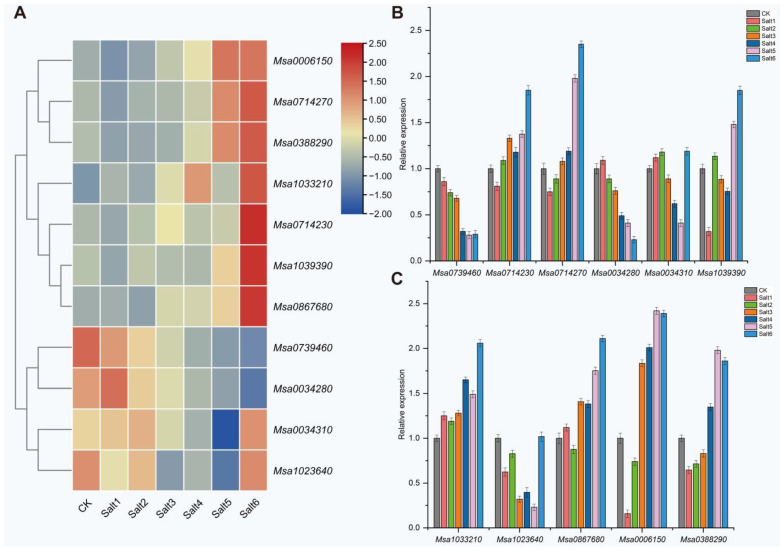
(**A**) Heatmap of the 11 DEGs that responded to salt stress. The expression levels were normalized by row using the Z-scores algorithm. The color scale on the right of the heatmap refers to the relative expression level, and the color gradient from blue to red presents an increasing expression level. (**B**) RT-qPCR analysis of *Msa0739460*, *Msa0714230*, *Msa0714270*, *Msa0034280*, *Msa0034310*, and *Msa1039390*. (**C**) RT-qPCR of *Msa1033210*, *Msa1023640*, *Msa0867680*, *Msa0006150*, and *Msa0388290*. CK was arbitrarily set to 1. Error bars represent the standard deviations of three technical replicates.

In this GWAS study, 103 candidate genes were found to be associated with 37/49 significant SNPs. Combined with the RNA-seq results, 11 DEGs were found among these 103 genes (Table S9). Among the 11 genes associated with salt stress in the root, 1 gene was differentially expressed in the Salt1 and Salt4 groups, 7 genes were differentially expressed in the Salt5 group, and 8 genes were differentially expressed in the Salt6 group. No DEGs were found in the Salt2 and Salt3 groups. We combined the GWAS and RNA-seq to further identify candidate genes involved in salt resistance responses and, finally, identified 11 candidate genes. The primers were designed for real-time quantitative polymerase chain reaction (RT-qPCR) validation to verify the RNA-seq results (Table S10). The results of the RT-qPCR were consistent with those of the transcriptome (Figure 5). As shown in Figure 5A, 11 DEGs were responsive to salt stress. Out of the 11 DEGs, the expression of *Msa0739460* decreased in all six groups. Excluding *Msa0739460* and *Msa0034280*, the other DEGs were upregulated in the Salt6 group (Figure 5A,B).

## 3. Discussion

Research shows that seedling vigor under salt stress is an effective selection method for breeding programs [25]. At present, minimal research has been conducted on salt tolerance in alfalfa [19,26,27]. In this study, the genetic basis of the salt tolerance of alfalfa was analyzed using a GWAS, revealing many promising gene targets significant for cultivating new alfalfa varieties in saline and alkaline soils.

Salt stress is a major abiotic factor that restricts plant growth and productivity; it can significantly reduce the fresh and dry weights of aboveground parts [28]. The root system is the first part of the plant to perceive toxic ions in the soil, and can guarantee the subsequent normal growth of plants [29]. Thus, the genetic mechanisms underlying different root traits have recently become a hot research topic. More recently, GWAS-based explorations have identified some genes related to roots that allow plants to acclimate to soil nitrogen [30] and phosphate [31] contents, and that are involved in root growth responses to salt stress [32]. Other studies showed that the root activity reduced, the RL shortened, and the lateral roots of plants became reduced under stress, which, ultimately, affected the overall RL and plant height [33,34]. In the present study, the phenotypic differences at different NaCl concentrations indicated that salt stress significantly inhibited the root growth of alfalfa seeds during germination. Previous studies showed that the RL of barley and maize was significantly reduced under salt stress [35,36], which was consistent with our results. The formation and spatial distribution of LRs were the most important factors mediating soil exploration by plants. In the present study, we observed that the LRs of germinated seeds decreased significantly after the salt treatment, indicating that the LRs were inhibited, which might further lead to a delayed seedling growth. Further, the current results showed that most traits were highly correlated, suggesting that they might undergo similar molecular regulatory mechanisms under different degrees of salt stress.

The results demonstrated that the landrace and cultivated varieties exhibited a greater salt tolerance compared with the wild varieties, suggesting that some varieties with a good tolerance were selected for breeding during the process of artificial selection. In addition, we found that the varieties from America and Turkey also exhibited a better salt tolerance. This might have been related to the fact that most alfalfa varieties in Asia and the western United States were grown in saline–alkaline soils, because most of the higher-quality farmland was used for planting staple food crops. This results further indicated that selective breeding to improve salt tolerance and geographical origins might affect the allelic diversity in alfalfa. Further, the research could lead to a greater understanding of salt-tolerance-related genes, which could be used to improve salt tolerance in cultivated varieties.

Alfalfa is sensitive to salts in the soil, especially during germination. GWASs are effective in identifying such genes. Yu et al. used GBS technology to identify 36 molecular markers significantly related to salt tolerance using 198 materials under three salt treatments [37]. Liu et al. used natural populations to identify 42 markers related to dry weight and plant height traits that were significantly related to salt tolerance [38]. In this study, 49 significant SNPs with root development were found. However, no consistent loci were detected, indicating that these markers were independently related to their respective characteristics and that different genetic structures and molecular mechanisms were responsible for different root responses in alfalfa under salt stress. Further research on these mechanisms could provide the theoretical basis for cultivating salt-tolerant alfalfa.

Although GWASs can be used to identify the genes involved in abiotic stress responses in a wide range of crops [12,19], unresolved problems, such as false positives, can hinder our understanding of these processes. RNA-seq analysis has also become a tool for detecting gene expression [39]. A large number of DEGs can be obtained from transcriptome data. However, identifying potential key candidates can be challenging [40]. In recent years, GWASs and RNA-seq analyses have been integrated to predict candidate genes for traits related to defoliation [41], drought stress [42], and salt stress in alfalfa [43]. In this study, we identified 11 DEGs in six different growth stages, all of which played a role in the response of alfalfa to salt tolerance. A number of plant hormones play a role in salt stress responses, including jasmonic acid and abscisic acid (ABA) [44]. *AtHAD1* had an inhibitory effect on ABA responses and ABA-mediated tolerance in *A. thaliana*. In this study, we found that *Msa0034310*, encoding HAD (haloacid-dehalogenase-like hydrolase), was significantly correlated with the root development of alfalfa under salt stress, as the expression of *Msa0034310* in roots was upregulated under salt stress. In addition, we found that on chromosome one, the marker chr1__8903020 (*Msa0006150*) encoded betaine aldehyde dehydrogenase, which was necessary for glycine betaine biosynthesis and positively regulated plant responses to stress [45]. Therefore, the overexpression and the knockdown experiments with these genes were required to validate their functions in alfalfa salt stress responses.

In conclusion, we identified 49 SNP markers, distributed across all eight alfalfa chromosomes, through the use of a GWAS. In this study, 103 salt stress response genes were associated with significant markers related to salt tolerance in the roots of alfalfa during germination. Combining RNA-seq data, we identified 11 candidate genes that could be used for gene cloning and functional characterization, thus, helping improve our understanding of salt tolerance in alfalfa.

## 4. Materials and Methods

### 4.1. Plant Materials

The association mapping panel consisted of 220 alfalfa (*Medicago sativa* L.) varieties [46]. After the alfalfa seeds were surface sterilized, 250 mM of a NaCl solution was used to treat the seeds. The seeds were grown in a greenhouse at 24 °C (day)/20 °C (night) under a 16 h light/8 h dark photoperiod at a relative humidity of 70–80% for 1 week. The root tips were collected after the NaCl treatment for 0, 0.5, 1, 3, 6, 12, and 24 h, which were renamed CK, Salt1, Salt2, Salt3, Salt4, Salt5, and Salt6, respectively. Three replicates, each of which included five seedlings, were collected for each treatment. The samples were stored at −80 °C prior to the RT-qPCR experiments.

### 4.2. Salt Stress Treatment and Phenotyping

A total of 20 seeds of each variety were placed in a plastic Petri dish, and 5 mL of the respective NaCl solution (100, 150, and 200 mM) or water (0 mM) was added to each plate [37]. In total, the 100 mM salt concentration consisted of 5.85 g sodium chloride in 1 L of water, the 150 mM salt concentration consisted of 8.775 g sodium chloride in 1 L of water, and the 200 mM salt concentration consisted of 11.7 g sodium chloride in 1 L of water. For both control and salt treatments, all alfalfa seeds were laid out in a randomized complete-block design with three replications. The root traits were examined after the seeds no longer germinated (the tenth day). Four traits were measured at different concentrations: the number of LRs, RL, RV, and RD.

### 4.3. Phenotypic Data Analysis

The SSI (stress susceptibility index) was calculated for each trait to evaluate salt tolerance using the following formula:

(1) $$\mathrm{SSI}=\frac{\mathrm{Ys}/\mathrm{Yn}}{\mathrm{Ms}/\mathrm{Mn}}$$

where Ys and Yn are values for the measured traits of the plant under stress and without stress, respectively; Ms and Mn are the mean values of the measured traits across all plants in the given test under stress and without stress conditions, respectively [47]. The phenotypic correlations were analyzed using the TBtools software [48].

### 4.4. SNP Calling and Genome-Wide Association Studies

The young leaves of the plants were collected, and the total DNA of the leaves was extracted with a Nuclean plant Genomic DNA kit (Kangwei century, Taizhou, China), which was used to construct the sequencing library. Sequencing using the Illumina NovaSeq 6000 platform (Illumina, San Diego, CA, USA) to generate 150 bp paired-end reads and approximately 10 Gb sequencing data, with an average Q30 of 85%, was used for each accession [24]. The paired-end sequencing reads were mapped to the assembled Zhongmu-4 genome using the default parameters of BWA-MEM [49]. Approximately 29.6 million SNPs were detected with the BWA SAMtools VarScan pipeline, and then filtered using the vcftools v.0.1.16 software by following the criteria of a missing rate ≤ 10%, minimum average reading depth > 5, and minor allele frequency (MAF) > 0.05 [24]. The obtained high density of SNP data was used for the GWASs. The GWASs were performed using the C language v0.01 version of the Bayesian-information and Linkage-disequilibrium Iteratively Nested Keyway software [50]. The threshold for significantly associated loci was a logarithm of odds score ≥6. Manhattan plots were constructed using the R 4.0.3 version software.

### 4.5. RNA-Seq and Transcriptomic Analysis

Transcriptomic data for alfalfa plants exposed to salt treatments were collected from the NCBI database (SRR7160314–SRR7160315, SRR7160322–SRR7160331, SRR7160339–SRR7160341, SRR7160351–SRR7160352, and SRR7160354–SRR7160357) [51]. The obtained raw sequencing reads were filtered with the fastp software, and the clean reads were retained. The gene expression levels were calculated using the clean reads mapped on Zhongmu-4 reference haploid genomes with the HISAT2 software [52], and were then normalized to transcripts per kilobase million (TPM) via a script. The average genome comparison rate was 71.03%. The TPM value was used to estimate the gene expression level and the differentially expressed genes were obtained using DESeq with padj < 0.05 and |log2FC| ≥ 1 [53]. The TBtools software was used for data visualization [48].

### 4.6. Candidate Gene Analysis and RT-qPCR

Based on the LD, all genes within the 40 kb range of significant sites were identified using the reference genome of Zhongmu-4. The orthologues were identified by comparing them with the *A. thaliana* genome. The candidate genes were identified by combining the GWAS and transcriptomic results. The root tips of the plants were collected and the RNA was extracted using the MiniBEST RNA kit (TaKaRa, Beijing, China), according to the manufacturer’s instructions, and washed with the DNA incubation solution provided with the kit to remove the residual DNA in the genome. In total, 60μL RNA was extracted from each sample and used to construct the cDNA library. A quantitative real-time PCR (qRT-PCR) was constructed using the Taq Pro Universal SYBR qPCR Master Mix kit (vazyme, Nanjing, China) on the CFX96 Touch™ RT-PCR system (BioRad, Los Angeles, CA, USA). Three technical replicates were set for each sample. All primers used in this study are listed in Table S10, and the alfalfa *actin* gene was used for an internal control. The data were quantified with the 2^−(ΔΔCT)^ method [54]. The SPSS 26 version software was used for the analysis of variance (ANOVA). Data visualization was presented using the Origin 2019b version software.

## Figures and Tables

**Table 1:** Phenotypic variation for 4 root traits under 3 different salt concentrations, including lateral roots (LRs), root length (RL), root volume (RV), and root diameter (RD), among 220 accessions.

Trait	Median	Mean	Range	SD	Kurtosis	Skewness	CV
LR_100	1.12	1.31	0.24–3.80	0.78	0.77	1.06	0.59
RV_100	0.96	1.03	0.35–2.52	0.40	1.45	1.21	0.39
RL_100	0.94	1.14	0.21–4.19	0.71	1.12	1.74	0.63
RD_100	1.00	1.03	0.54–1.71	0.24	0.02	0.44	0.43
LR_150	0.91	1.37	0.21–4.32	1.12	0.33	1.20	0.82
RV_150	0.94	1.11	0.22–3.37	0.59	1.08	1.65	0.53
RL_150	0.92	1.02	0.32–2.57	0.50	0.70	1.09	0.49
RD_150	1.02	1.04	0.50–1.72	0.28	−0.60	0.20	0.37
LR_200	0.91	1.21	0.25–4.95	0.99	4.23	1.93	0.81
RV_200	0.88	0.91	0.27–1.93	0.29	1.44	0.76	0.32
RL_200	1.04	1.06	0.64–1.58	0.26	−0.95	0.30	0.44
RD_200	1.11	1.72	0.31–5.57	1.48	0.51	1.30	0.86

## Data Availability

Not applicable.

## Supplementary Materials

The following supporting information can be downloaded at: https://www.mdpi.com/article/10.3390/ijms24076271/s1.
