# Supplementary material for: Transcriptome and GWAS Analyses Reveal Candidate Gene for Root Traits of Alfalfa during Germination under Salt Stress
Source: Int J Mol Sci. 2023 Mar 27;24(7):6271. doi: 10.3390/ijms24076271 (PMC10094355; doi:10.3390/ijms24076271)
Supplement: Supplementary file 1 [file ijms-24-06271-s001.zip › ijms-2203350-supplementary captions.pdf]

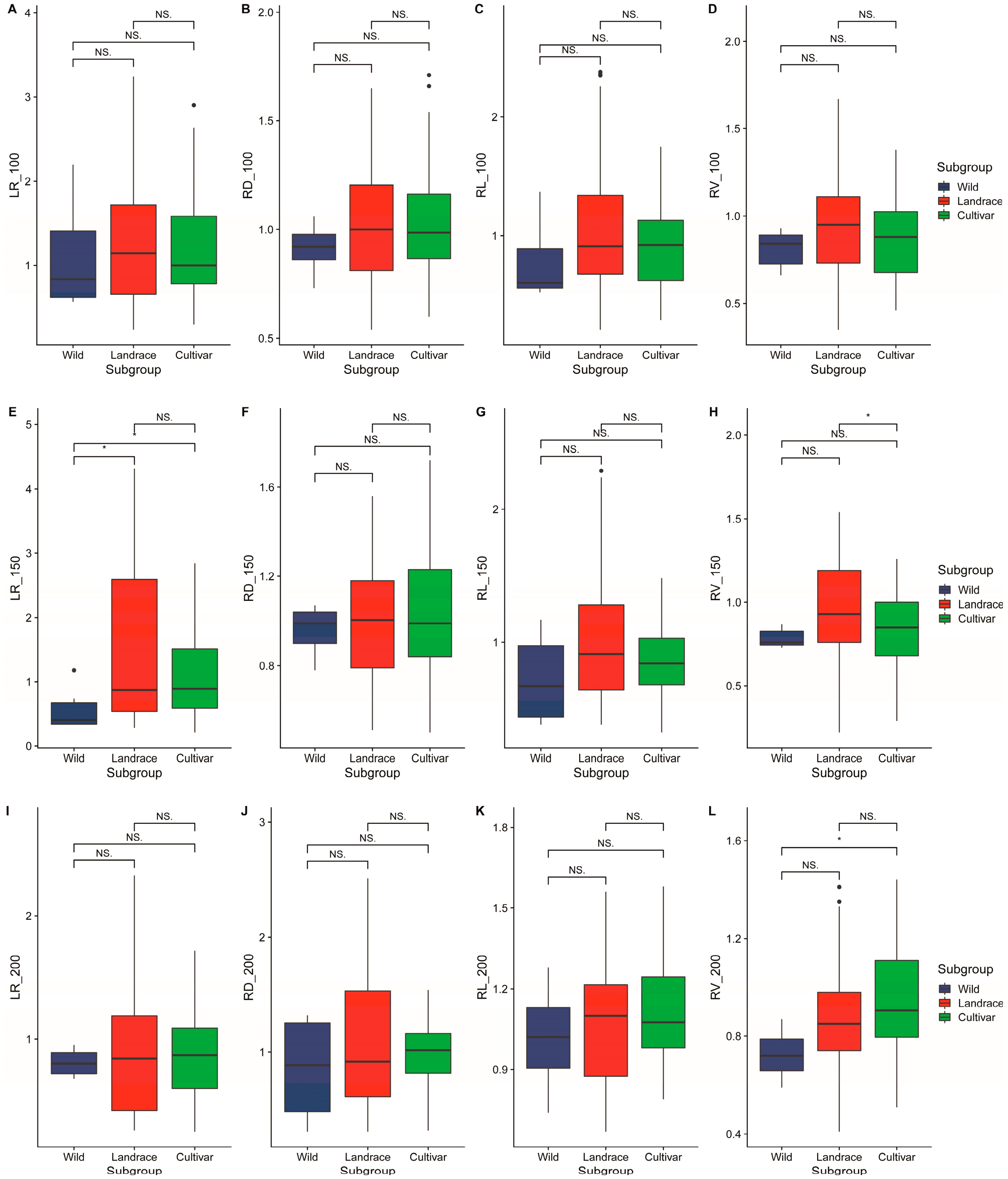

Figure S1. Box plot of the tolerance to the salt stress among subgroup (Wild, Landrace and Cultivar) under three salt concentrations according to breeding status. A-D: Boxplot of each root character at 100mM salt concentrations. E-H: Boxplot of each root character at 150mM salt concentrations. I-L: Boxplot of each root character at 200mM salt concentrations. *, indicate significance at $p < 0.05$.

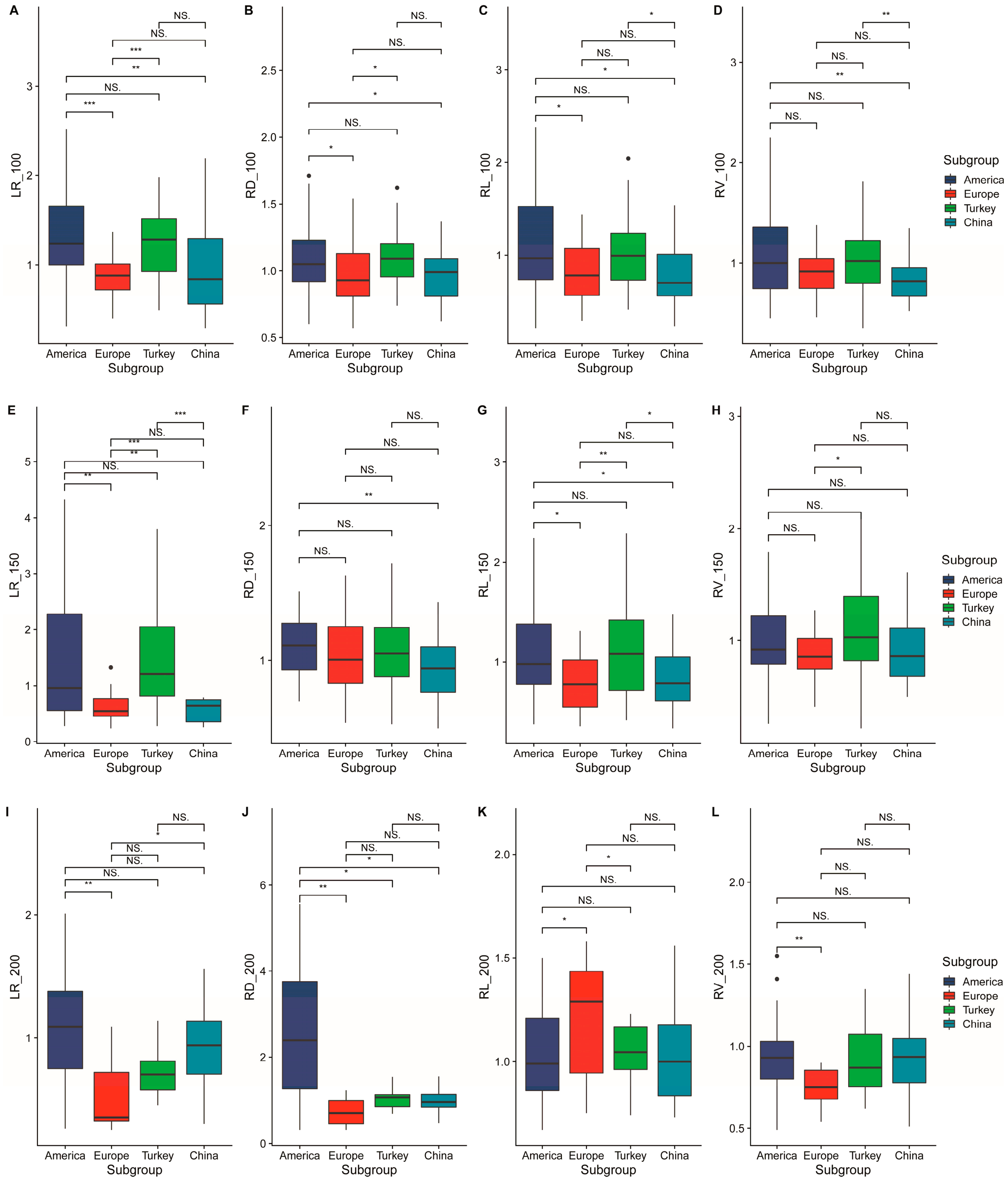

Figure S2. Box plot of the tolerance to the salt stress among subgroup (Europe, Asia, America and Africa) under three salt concentrations according to geographical origin. A-D: Boxplot of each root character at 100mM salt concentrations. E-H: Boxplot of each root character at 150mM salt concentrations. I-L: Boxplot of each root character at 200mM salt concentrations. *, **, and *** indicate significance at $p < 0.05$, $p < 0.01$, and $p < 0.001$, respectively.

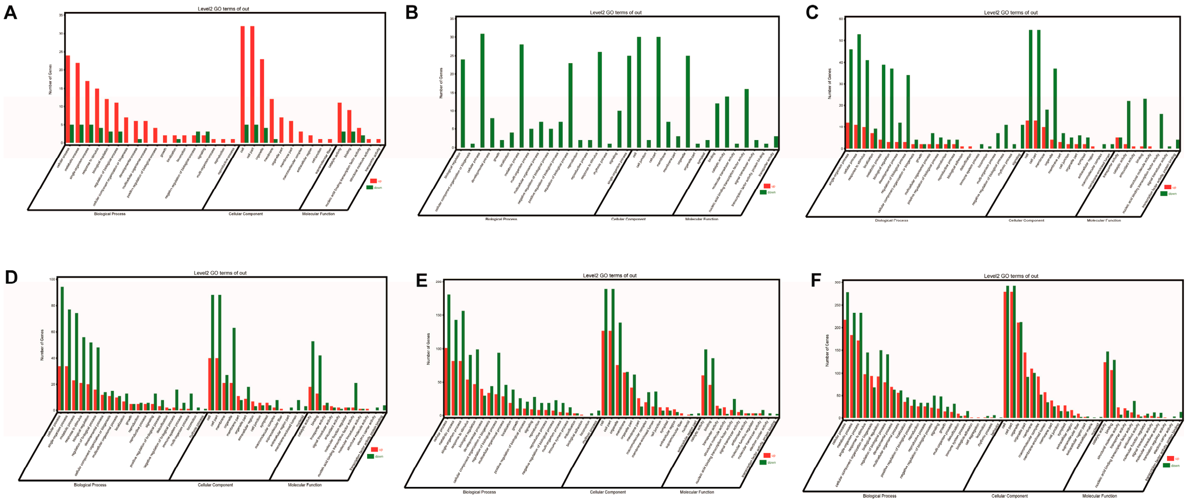

Figure S3. GO analysis of all DEGs. A–F: GO analysis of the Salt1 - Salt6.
